# Margaret Buckingham, discoveries in skeletal and cardiac muscle development, elected to the National Academy of Science

**DOI:** 10.1186/2044-5040-2-12

**Published:** 2012-06-07

**Authors:** Michael A Rudnicki

**Affiliations:** 1 Ottawa Hospital Research Institute, Ottawa, Ontario, K1H 8L6, Canada

**Keywords:** National Academy of Sciences, Cardiac development, Skeletal muscle development

## Abstract

Margaret Buckingham was presented as a newly elected member to the National Academy of Sciences on 28 April 2012. Over the course of her career, Dr Buckingham made many seminal contributions to the understanding of skeletal muscle and cardiac development. Her studies on cardiac progenitor populations has provided insight into understanding heart malformations, while her work on skeletal muscle progenitors has elucidated their embryonic origins and the transcriptional hierarchies controlling their developmental progression.

## **Commentary**

Dr Margaret Buckingham, a much-respected investigator who has made many significant contributions to our understanding of skeletal muscle and cardiac development, was elected to the National Academy of Sciences in 2011 and presented on 28 April, 2012. Dr Buckingham is Professor in the Department of Developmental Biology at the Pasteur Institute in Paris. She has been awarded many prestigious distinctions including that of *Officier de la Légion d’Honneur* and *Officier de l'Ordre National du Mérite*, to name but two.

Dr Buckingham’s early studies involved the cloning and characterization of actin and myosin genes from cardiac and skeletal muscle [1-4]. She has made seminal contributions to our understanding of cardiac development. She identified the second heart field [5] and showed its important contribution to the poles of the heart. These cardiac progenitors are regulated by a distinct genetic network and in this context, Dr Buckingham has worked on the role of fibroblast growth factor (FGF) signaling in the formation of the outflow tract and pharyngeal arteries [6]. Her studies of cardiac development revealed that that two cell lineages contribute to the myocardium [7]. Lineage studies also demonstrated a clonal relationship between arterial pole myocardium and head muscles [8]. Her work on cardiac progenitor populations is of clinical importance in understanding heart malformations.

Dr Buckingham has also made major contributions to the molecular genetic analysis of skeletal muscle development. She was the first to analyze expression of the myogenic regulatory factors of the MyoD family during mouse embryogenesis [9] and the behavior of cells in the absence of Myf5 [10].

Her more recent work demonstrated that skeletal muscle growth depends on a somite-derived population of progenitor cells that express Pax3 and Pax7 [11]. She established that the *Myf5* gene is activated by Pax3 through specific regulatory elements [12], and that Pax3 regulation of FGF signaling affects the balance between progenitor self-renewal and differentiation [13]. She showed genetically that before cells acquire myogenic potential, the equilibrium between *Pax3* and *Foxc2* expression in the somite regulates the choice between myogenic versus vascular cell fate [14]. After demonstrating the key role of satellite cells in adult muscle regeneration [15], she investigated satellite-cell quiescence, showing recently that microRNA31 targets *Myf5* mRNA, and that both are sequestered in micro-ribonucleoprotein granules which breakdown on satellite cell activation [16]. She also identified microRNA-27 as a regulator of Pax3 production [17].

Dr Buckingham’s election to the academy recognizes her many significant contributions as a leading scholar in the molecular genetic study of striated muscle formation.

## **Competing interests**

The author declares no competing interests.
